# Unequally Distributed Psychological Assets: Are There Social Disparities in Optimism, Life Satisfaction, and Positive Affect?

**DOI:** 10.1371/journal.pone.0118066

**Published:** 2015-02-11

**Authors:** Julia K. Boehm, Ying Chen, David R. Williams, Carol Ryff, Laura D. Kubzansky

**Affiliations:** 1 Department of Psychology, Chapman University, Orange, California, United States of America; 2 Department of Social and Behavioral Sciences, Harvard School of Public Health, Boston, Massachusetts, United States of America; 3 Department of African and African American Studies and Sociology, Harvard University, Cambridge, Massachusetts, United States of America; 4 Department of Psychology, University of Wisconsin Madison, Madison, Wisconsin, United States of America; 5 Institute on Aging, University of Wisconsin Madison, Madison, Wisconsin, United States of America; Merced, UNITED STATES

## Abstract

Socioeconomic status is associated with health disparities, but underlying psychosocial mechanisms have not been fully identified. Dispositional optimism may be a psychosocial process linking socioeconomic status with health. We hypothesized that lower optimism would be associated with greater social disadvantage and poorer social mobility. We also investigated whether life satisfaction and positive affect showed similar patterns. Participants from the Midlife in the United States study self-reported their optimism, satisfaction, positive affect, and socioeconomic status (gender, race/ethnicity, education, occupational class and prestige, income). Social disparities in optimism were evident. Optimistic individuals tended to be white and highly educated, had an educated parent, belonged to higher occupational classes with more prestige, and had higher incomes. Findings were generally similar for satisfaction, but not positive affect. Greater optimism and satisfaction were also associated with educational achievement across generations. Optimism and life satisfaction are consistently linked with socioeconomic advantage and may be one conduit by which social disparities influence health.

## Introduction

Disparities in health and longevity based on socioeconomic status (SES) are well established [1]. Individuals with lower social status have greater risk of disease and mortality relative to individuals with higher social status. However, less well understood are the pathways underlying this association. Access to health care, engaging in preventative health behaviors like exercising, and exposure to environmental hazards have been suggested as pathways by which SES and health are connected. Psychosocial processes have also been theorized as a possible pathway by which SES and health are linked. For example, the reserve capacity framework posits that individuals in low socioeconomic contexts are exposed to more stressful situations, which may strain the psychosocial resources (e.g., social support, feelings of control, optimism) that can be used for managing challenges [2]. Moreover, repeated encounters with stress may reduce the number of opportunities that individuals with low SES have to either develop or replenish their psychosocial resources [3]. With both more stressful experiences and fewer psychosocial resources with which to cope, worse health outcomes may ensue [4].

Dispositional optimism—or the generalized expectation that good rather than bad events will occur [5]—has been characterized as a health asset. In other words, optimism can be considered a psychosocial resource that may protect health over the life course [6,7]. This is based in part on a growing body of research indicating that optimistic individuals have reduced risk of heart disease and all-cause mortality compared with less optimistic individuals [8–10]. Several investigators have also reported that optimism appears to be associated with social status, with findings in non-U.S. cohorts suggesting higher optimism is associated with higher SES [11,12]. As a result, optimism has been put forward as a potential psychosocial resource that may explain in part why higher social status is associated with better health [4,13–15].

Optimism can serve as a mechanism by which social advantage leads to better health only if it is itself systematically patterned and produced by income, education, and race/ethnicity such that those with greater social advantage have higher levels of optimism. However, although optimism is only partly heritable [16], relatively little is known about its distribution across the social structural factors that are so strongly linked to health disparities—hereafter called structural factors [5]. This is partly because epidemiological cohorts do not typically assess dispositional optimism; they more often assess other psychological assets (i.e., indicators of positive psychological functioning) such as life satisfaction and positive affect. Limited research indicates greater optimism is associated with higher SES (most often indicated by education) among older community members [12] and adolescents or young adults [13,17,18]. But no research has systematically examined optimism’s association with key structural factors contributing to social disparities in health among U.S. adults including race/ethnicity, education, occupational class and prestige, and income.

In addition to prior work in British and Finnish cohorts suggesting that low SES is associated with viewing the future less optimistically [11,12], research with other indicators of psychological health suggests that while they may seem intensely personal, they are in fact patterned by structural factors. For example, low SES individuals are more likely to experience higher levels of depression and anxiety with greater frequency [19–21]. Moreover, life satisfaction is associated with social advantage [22,23] such that greater satisfaction is linked with more education and higher income [24–27]. Whites also tend to report being more satisfied than Blacks and Hispanics in the U.S. [26]. In sum, prior work indicates substantial social disparities in some of the most commonly assessed indicators of psychological health.

Thus, there is reason to suspect that dispositional optimism is similarly patterned by the structural factors that influence access to resources and are linked with health disparities. We posit that optimism may be associated with disparities-related factors because these factors provide a context for either developing or restricting optimistic tendencies. For example, optimistic people expect favorable outcomes, persist at goals, use effective coping strategies, and engage with attainable goals or disengage from unattainable goals [28]. Higher levels of education may foster such skills, as well as the opportunity for positive feedback loops to develop about goal attainment that validate optimistic perspectives. By contrast, poverty and/or the experience of being a racial or ethnic minority may suppress optimistic tendencies because individuals encounter more demanding environments and have fewer resources to combat challenges [20]. Because they are more frequently exposed to unpredictable and difficult situations, individuals of low SES are more likely to develop a schema that the world is threatening, and therefore interpret ambiguous events with more negative and fewer positive attributions [29]. Such experiences are unlikely to routinely foster upward spirals of optimism. Notably, SES is frequently perpetuated across generations [30,31], and this may extend to the corresponding cognitive and affective processes that are associated with lower social status [20]. Thus, in addition to understanding optimism’s association with structural factors, it is also useful to consider whether optimism is associated with the educational attainment of one’s parents or responsive to social mobility.

Taken together, our primary aim was to investigate dispositional optimism’s association with a set of social structural factors related to health disparities: gender, race/ethnicity, education level (of the participant and his or her parents), occupational class, occupational prestige, and income. Consistent with previous research, we expected that higher social position (e.g., higher levels of education and income) would be associated with greater optimism. We also considered age-related patterning as health-related social disparities widen with age [32]. For comparative purposes, and because satisfaction and positive affect are more often included in large epidemiological cohorts, we also considered whether social patterning in optimism is similar to patterning with these psychological assets. Although numerous studies have considered the distribution of life satisfaction or positive affect across social indicators, few have compared these patterns with those for optimism. Given the moderate to high correlations between these psychological assets [33], we expected them to be similarly distributed. However, evidence that they are not similarly distributed might suggest that unique psychological assets are not equally relevant for understanding how social disadvantage translates into health outcomes.

A secondary aim was to use a life course perspective to investigate the association of each psychological asset with intergenerational continuity and change in educational attainment. Evidence from a Finnish cohort suggested that optimism was associated with intergenerational trajectories of social mobility such that individuals with persistently high SES (present in childhood and adulthood) were more optimistic than individuals with persistently low SES or who became upwardly or downwardly mobile [11]. We sought to replicate that finding with educational achievement in U.S. participants, and to examine whether satisfaction and positive affect were similarly influenced by intergenerational trajectories. To our knowledge, limited research has considered whether different psychological assets are influenced by social mobility.

## Methods

### Participants

Participants were men and women from the Midlife in the United States (MIDUS) study. MIDUS was initially designed to investigate behavioral, psychological, and social factors in aging individuals. The first phase (MIDUS I) began in 1995 and included a national random-digit-dialing sample, oversamples from five metropolitan areas, siblings of the random-digit-dialing sample, and a national random-digit-dialing sample of twin pairs (total *N* = 7,108). Individuals were eligible to participate if they were non-institutionalized, spoke English, and were 25–74 years old. A longitudinal follow-up began an average of 9 years later in 2004 (MIDUS II); 4,963 of the original MIDUS I respondents participated in MIDUS II [34]. A new sample of Blacks was also recruited from Milwaukee to participate in MIDUS II (*N* = 592). Our analyses were based on MIDUS II data so Milwaukee participants could be included and because a well-validated measure of dispositional optimism was assessed then. Thus, the initial sample of 5,555 included main MIDUS II and Milwaukee participants.

The analytic sample for the current analyses was further reduced by 1,140 participants missing data on optimism, life satisfaction, and positive affect. The three psychological assets were assessed with self-administered questionnaires returned by mail, so fewer participants completed them compared with the primary phone interview. However, among participants who completed the measures of psychological assets, the vast majority completed all items and over 98% completed at least two thirds of the relevant items in each measure (n = 4,415). Among these participants, some were missing data on structural factors. Where possible, missing data on structural factors at MIDUS II was replaced with MIDUS I data. Thus, sample sizes in the primary analyses were 3,844–4,415, with 1,837 members of a sibling or twin set in the analytic sample. Included versus excluded participants tended to be women who were older, white, educated, of a higher occupational class, and with higher household incomes. As a result, estimates of social disparities in the psychological assets may be conservatively estimated in this sample. All respondents provided written informed consent. Original data collection was approved by institutional review boards at the University of Wisconsin Madison, University of California Los Angeles, and Georgetown University. Secondary data analyses were approved by the institutional review boards at Chapman University and Harvard School of Public Health.

### Measures

**Social structural factors**. Social structural factors were self-reported by participants at MIDUS II except for parental education, which was self-reported at MIDUS I for non-Milwaukee participants. If social structural information from MIDUS II was not available, information from MIDUS I was used when possible. The social structural factors included gender (men, women), race/ethnicity (white, black; other races were excluded), highest level of education attained by either the participant’s mother or father, and highest level of education attained by the participant (each coded as less than high school, high school diploma, some college, college degree or more). In addition, we assessed occupational class (managerial/professional, technical/sales/clerical/service, manual; homemakers and individuals not currently working were excluded if their previous job was unknown) and Duncan’s socioeconomic index (SEI) as an indicator of the status of participants’ current job or previous job if retired [35]. SEI was standardized (M = 0, SD = 1). Standardized scores of +1 (e.g., public administrators with SEI = 54.35), 0 (e.g., postmaster with SEI = 39.84), and-1 (e.g., apparel salesperson with SEI = 25.37) approximated high, average, and low prestige occupations, respectively [36]. Finally, we assessed household income in U.S. dollars, which was further categorized into quintiles ($0 to <$21,000, $21,000 to <$40,250, $40,250 to <$63,750, $63,750 to <$102,750, $102,750 to $300,000 or more). To maintain confidentiality, household incomes greater than $300,000 were converted to $300,000.

Following previous work [11,37], we created four trajectories of social mobility across generations for the 1,959 participants whose parents had either less than a high school diploma or a college degree or more. We excluded participants whose parents were moderately educated to focus on effects of either upward or downward social mobility, or stable SES from extreme social-class origins. Intergenerational trajectories of education were categorized as persistently high (high parental education and participant education; *n* = 592, 30.22%), downwardly mobile (high parental education and low participant education; *n* = 292, 14.91%), upwardly mobile (low parental education and high participant education; *n* = 932, 47.58%), and persistently low (low parental education and participant education; *n* = 143, 7.30%).

**Psychological assets**. Dispositional optimism was defined as generally having favorable expectations for the future. Although this is the most common way to define optimism, other definitions exist. These include, for example, explanatory style optimism and pessimism (i.e., the pattern of attributions that individuals make about events in their lives) [38], unrealistic or comparative optimism (i.e., an individual’s belief that he or she is more likely to encounter positive events and less likely to encounter negative events than others) [39], and situation-specific optimism (i.e., having favorable expectations for a specific event) [40]. Despite using the term “optimism”, these three other definitions are weakly to moderately correlated with dispositional optimism and are considered distinct constructs [5,39,40]. In this study, dispositional optimism was assessed with the valid and reliable six-item Life Orientation Test-Revised [41]. Example items are “I expect more good things to happen to me than bad” and “I hardly ever expect things to go my way” (1 = *agree a lot* to 5 = *disagree a lot*). Although past work has occasionally derived subscales from the three positively-worded (α = .68) and the three negatively-worded items (α = .80; in the present study, the subscales were correlated *r* = -.44), we followed recommendations to use all six items to more fully characterize optimism [42,43] and to achieve higher internal consistency. An optimism score was derived by reverse coding items as necessary and then summing responses such that greater numbers reflected greater optimism (possible scores ranged from 6–30). Following MIDUS data conventions, if at least three items were completed, a missing value was imputed with the mean value of the other items. Internal consistency reliability in the present sample was good (α = .79).

Life satisfaction, defined as judgments about life in general or specific life domains [44], was assessed with six previously validated items [45,46] that asked participants to rate their satisfaction with work, health, relationship with spouse/partner, relationship with children, financial situation, and life overall (0 = *the worst possible* to 10 = *the best possible*). The two items regarding relationship satisfaction were first averaged together and subsequently averaged with the remaining items for an overall life satisfaction mean [46]. Following MIDUS data conventions, if at least one item was completed, a mean score was computed with greater numbers reflecting greater satisfaction (possible scores ranged from 1–10). Internal consistency reliability in the present sample was acceptable (α = .72).

Positive affect (colloquially known as happiness) was assessed in response to six items referring to the question: “During the past 30 days, how much of the time did you feel…” Items included “cheerful”, “in good spirits”, “extremely happy”, “calm and peaceful”, “satisfied”, and “full of life” (1 = *all of the time* to 5 = *none of the time*) [47]. Following MIDUS data conventions, a mean positive affect score was computed if at least one of the six items was completed. Greater scores reflected more positive affect (possible scores ranged from 1–5). Internal consistency reliability in the present sample was excellent (α = .91). We also examined positive affect assessed with the Positive Affect and Negative Affect Schedule [48] and the Multidimensional Personality Questionnaire [49,50], but patterns were similar so results are not discussed.

### Statistical Analyses

Descriptive analyses were first conducted. Differences in mean psychological assets according to each individual structural factor were then examined with analyses of variance (ANOVAs), which indicate whether at least one level of social strata differs from the others. We also conducted focused contrast analyses that tested a linear trend to reflect the expected social gradient [51]. These contrast analyses yielded *F*_contrast_ (which provides the significance level for the linear trend) and *r*_contrast_ (which provides the magnitude of effect for the linear trend). Multivariate models with all structural factors simultaneously predicting the individual psychological assets were also conducted. The psychological assets were also examined in relation to social mobility trajectories using ANOVAs. Because of sibling and twin pairs in the data, we conducted generalized estimating equations for primary analyses. Findings were nearly identical (data not shown), suggesting that clustering in the data did not bias parameter estimates. We present the unadjusted findings for easier interpretation.

## Results

### Sample characteristics

Participants were men (44%) and women (56%), ages 30–85 years (*M* = 55.85, *SD* = 12.37). Participants tended to be white, have a high school education or more, be employed in managerial/professional or technical/sales/clerical/service positions, and have an average level of occupational prestige (Table 1). Younger individuals reported the lowest levels of optimism, satisfaction, and positive affect relative to older individuals (Table 2). However, in general, participants tended to report levels of psychological assets above the midpoint on each rating scale (optimism *M* = 23.03, *SD* = 4.75; satisfaction *M* = 7.44, *SD* = 1.32; positive affect *M* = 3.44, *SD* = 0.72). For example, the average participant experienced positive affect some or most of the time. The three psychological assets were correlated (optimism and satisfaction *r* = .44; optimism and positive affect *r* = .44; satisfaction and positive affect *r* = .52).

**Table 1:** Sample characteristics.

Characteristic	Number	Percentage
**Age**		
30–39	415	9.40%
40–49	1103	24.98%
50–59	1246	28.22%
60–69	931	21.09%
70–85	720	16.31%
**Gender**		
Women	2485	56.29%
Men	1930	43.71%
**Race/ethnicity**		
Black	533	12.61%
White	3694	87.39%
**Highest level of parental education**		
Less than high school degree	1075	26.19%
High school degree	1498	36.50%
Some college	647	15.77%
College degree or more	884	21.54%
**Highest level of participant education**		
Less than high school degree	326	7.38%
High school degree	1223	27.70%
Some college	1278	28.95%
College degree or more	1588	35.97%
**Occupational class**		
Manual	768	19.98%
Technical/sales/clerical/service	1494	38.87%
Managerial/professional	1582	41.16%
**Occupational prestige**		
Low	739	17.19%
Average	2848	66.26%
High	711	16.54%
**Household income**		
$0-$20,500	857	19.59%
$21,000-$40,000	809	18.50%
$40,250-$63,500	877	20.05%
$63,750-$102,500	936	21.40%
$102,750-$300,000	895	20.46%

*Note*. Sample size for each analysis ranged from 3,844 to 4,415 participants. To maintain confidentiality, household incomes greater than $300,000 were converted to a maximum value of $300,000

**Table 2:** Social disparities in psychological assets.

	Psychological Assets											
	Optimism				Life Satisfaction				Positive Affect			
Characteristic	Mean (*SD*)	Omnibus *F*	Contrast *F*	Contrast *r* (95% CI)	Mean (*SD*)	Omnibus *F*	Contrast *F*	Contrast *r* (95% CI)	Mean (*SD*)	Omnibus *F*	Contrast *F*	Contrast *r* (95% CI)
**Age**		22.17***	44.18***	.10		46.22***	102.09***	.15		26.93***	50.60***	.11
				(.07-.13)				(.12-.18)				(.08-.14)
30–39	22.01 (4.79)				7.23 (1.26)				3.35 (0.71)			
40–49	22.35 (5.03)				7.15 (1.32)				3.33 (0.74)			
50–59	23.11 (5.02)				7.32 (1.37)				3.39 (0.74)			
60–69	24.06 (4.36)				7.77 (1.23)				3.61 (0.66)			
70–85	23.20 (3.96)				7.78 (1.18)				3.54 (0.69)			
**Gender**		0.85	0.85	.01		0.08	0.08	.004		1.01	1.01	.02
				(-.02-.04)				(-.03-.03)				(-.01-.04)
Women	22.98 (4.89)				7.43 (1.34)				3.43 (0.74)			
Men	23.11 (4.57)				7.45 (1.28)				3.45 (0.70)			
**Race/Ethnicity**		17.41***	17.41***	.06		105.68***	105.68***	.16		36.50***	36.50***	.09
				(.03-.09)				(.13-.19)				(0.06-.12)
Black	22.28 (4.64)				6.91 (1.57)				3.62 (0.81)			
White	23.20 (4.76)				7.53 (1.26)				3.42 (0.70)			
**Parental Education**		12.42***	6.04**	.04		1.76	0.22	.007		5.76***	0.06	.004
				(.008-.07)				(-.02-.04)				(-.03-.03)
Less than high school degree	22.79 (4.57)				7.51 (1.33)				3.49 (0.71)			
High school degree	22.84 (4.83)				7.42 (1.29)				3.39 (0.74)			
Some college	23.71 (4.74)				7.51 (1.26)				3.48 (0.68)			
College degree or more	23.78 (4.67)				7.51 (1.21)				3.40 (0.66)			
**Participant Education**		89.25***	40.87***	.10		33.17***	8.94**	.04		0.43	0.53	.01
				(.07-.12)				(.02-.07)				(-.02-.04)
Less than high school degree	20.51 (4.49)				7.00 (1.50)				3.46 (0.84)			
High school degree	22.03 (4.65)				7.34 (1.40)				3.43 (0.74)			
Some college	23.12 (4.72)				7.36 (1.32)				3.44 (0.71)			
College degree or more	24.26 (4.53)				7.67 (1.16)				3.45 (0.68)			
**Occupational Class**		67.87***	70.52***	.13		24.83***	32.90***	.09		0.10	0.20	.007
				(.10-.17)				(.06-.12)				(-.02-.04)
Manual	22.05 (4.46)				7.39 (1.26)				3.48 (0.72)			
Technical/sales/clerical/service	22.82 (4.67)				7.46 (1.20)				3.48 (0.71)			
Managerial/professional	24.21 (4.55)				7.70 (1.13)				3.47 (0.67)			
**Occupational Prestige**		74.54***	81.09***	.14		47.32***	50.67***	.11		1.74	0.74	.01
				(.11-.17)				(.08-.14)				(-.02-.04)
Low	21.40 (4.84)				7.08 (1.45)				3.45 (0.79)			
Average	23.14 (4.69)				7.46 (1.30)				3.43 (0.72)			
High	24.36 (4.43)				7.74 (1.10)				3.48 (0.65)			
**Household Income**		34.75***	135.03***	.17		58.62***	228.04***	.22		0.66	1.82	.02
				(.14-.20)				(.19-.25)				(-.009-.05)
$0-$20,500	21.83 (4.90)				6.97 (1.65)				3.41 (0.81)			
$21,000-$40,000	22.48 (4.75)				7.31 (1.30)				3.44 (0.70)			
$40,250-$63,500	23.08 (4.73)				7.43 (1.26)				3.44 (0.73)			
$63,750-$102,500	23.32 (4.63)				7.59 (1.10)				3.44 (0.68)			
$102,750-$300,000	24.32 (4.41)				7.86 (1.03)				3.47 (0.67)			

*Note*. Sample size for each analysis ranged from 3,844 to 4,415 participants. To maintain confidentiality, household incomes greater than $300,000 were converted to a maximum value of $300,000. Abbreviations: CI = confidence interval; *SD* = standard deviation. The omnibus *F* indicates whether at least one level of social strata differs significantly from the others. The contrast *F* and *r* provide the significance level and magnitude of effect, respectively, for the linear trend reflecting a social gradient. Statistically significant *F* values are indicated with

* *p* ≤. 05

** *p* ≤. 01

*** *p* ≤. 001

### Social disparities in psychological assets

The social structural patterning of the three psychological assets is shown in Table 2. Men and women did not differ in reported levels of optimism, satisfaction, or positive affect. The psychological assets were associated with race/ethnicity. Optimism and life satisfaction were significantly higher among Whites relative to Blacks. In contrast, Blacks reported higher positive affect relative to Whites. Optimism also seemed to be strongly patterned by other SES indicators. For example, optimism’s association with education in one’s family of origin was robustly graded such that participants who had a parent with a college degree or more were the most optimistic compared to those who had a parent with a high school diploma or less. However, satisfaction did not vary based on parental education. Moreover, a social gradient was not evident for positive affect in the context of parental education. Counter to expectation, participants whose most highly educated parent did not complete high school reported the highest levels of positive affect. Participants’ own education level was significantly and positively associated with optimism and satisfaction. Individuals with a college degree or higher reported being the most optimistic and satisfied compared with less educated individuals. For optimism in particular, the association was strikingly linear such that with each increase in education level, there was an associated increase in optimism. By contrast, positive affect was not significantly associated with participants’ level of education.

The highest levels of optimism and satisfaction were evident among individuals with managerial or professional occupations (i.e., the highest occupational class). Furthermore, both optimism and satisfaction had a linear relationship with occupational class such that manual occupations (i.e., the lowest occupational class) had the lowest levels and managerial occupations (i.e., the highest occupational class) had the highest levels. By contrast, positive affect did not differ by occupational class and a linear trend was not evident. Greater occupational prestige was also linearly associated with higher levels of optimism and satisfaction, but positive affect did not differ by occupational prestige. A similar pattern was evident for income. Optimism and satisfaction had a linear association with income such that individuals with the highest income reported the greatest optimism relative to their less wealthy counterparts. Positive affect was not significantly associated with income.

Multivariate models that included all of the structural factors simultaneously showed nearly identical patterns as the univariate models presented in Table 2 (data not shown). The percent of variance explained by all structural factors was 9% for optimism, 12% for satisfaction, and 4% for positive affect.

### Trajectories of social mobility across generations

**Fig 1:**
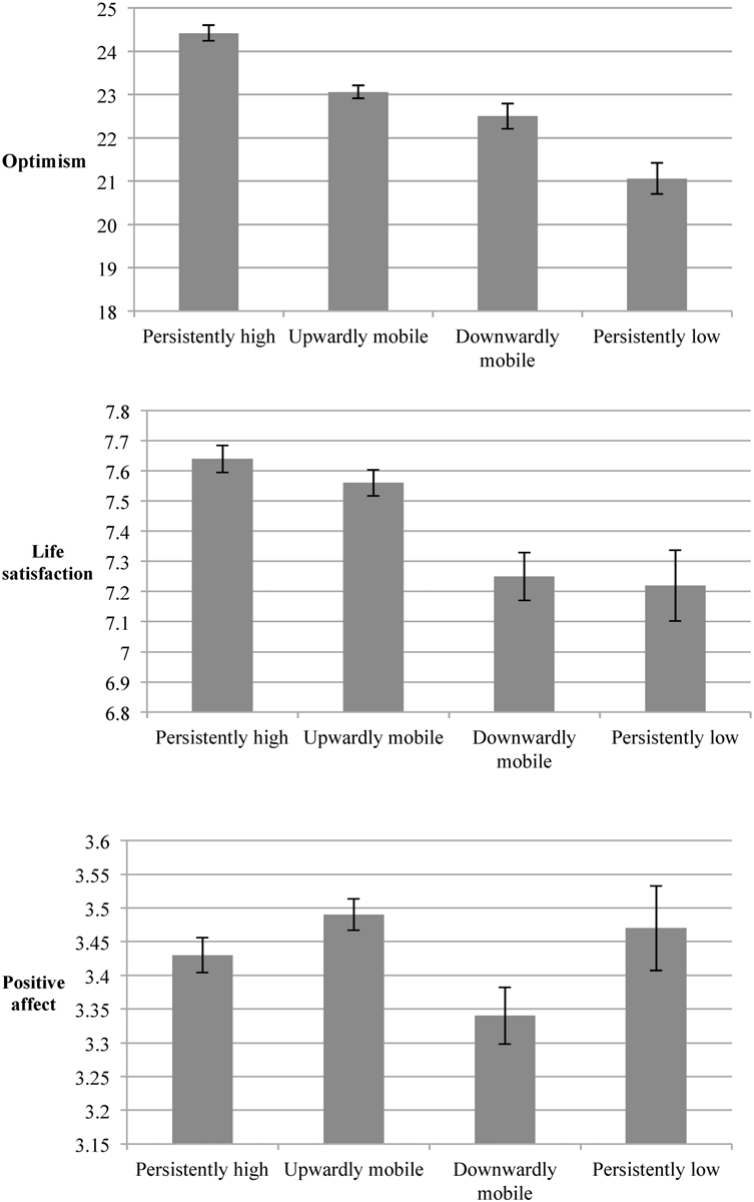
Means and standard errors of psychological assets according to intergenerational educational attainment (*N* = 1,959). Optimism is depicted in the top panel, life satisfaction is depicted in the middle panel, and positive affect is depicted in the bottom panel. For optimism, all pairwise comparisons were significantly different except the comparison between the upwardly mobile and the downwardly mobile. For satisfaction, all pairwise comparisons were significantly different except the comparisons between the persistently high and upwardly mobile, as well as the persistently low and the downwardly mobile. For positive affect, only the comparison between the upwardly mobile and the downwardly mobile was significantly different. *Note*. Only participants with low or high parental education were included in these analyses (i.e., participants whose parents were moderately educated were excluded).

Different patterns of optimism, life satisfaction, and positive affect emerged for trajectories of social mobility across generations (Fig. 1). Individuals with persistently higher education across generations had significantly greater optimism than the upwardly mobile, downwardly mobile, and persistently lower educated (post-hoc comparisons *p* < 0.05). Individuals with persistently lower education were the least optimistic relative to the other groups (post-hoc comparisons *p* < 0.05). Satisfaction demonstrated a similar pattern with the persistently higher educated individuals reporting significantly greater satisfaction compared with all other trajectories except for the upwardly mobile (post-hoc comparisons *p* < 0.05). Positive affect differed significantly between individuals who were upwardly versus downwardly mobile across generations such that the upwardly mobile reported greater positive affect (post-hoc comparison *p* < 0.05). No other comparisons were statistically significant for positive affect.

## Discussion

Although optimism is relevant for health outcomes [6,7,9,10] and is an important psychosocial asset in its own right [5], little is known about its social distribution, particularly among U.S. adults. We examined dispositional optimism’s social structural patterning in the context of a social disparities framework and compared findings on optimism with two more commonly assessed measures of psychological assets in epidemiological cohorts: life satisfaction and positive affect. Our hypothesis that social disparities in optimism would be evident was largely supported (although associations were modest), which is consistent with the reserve capacity framework [4]. Greater levels of optimism were evident among more socially advantaged individuals—that is, those without minority status who had more education, higher occupational classes and prestige, and larger incomes.

Consistent with previous findings [25], optimism, satisfaction, and positive affect did not differ between men and women. However, the patterning across other social structural factors tended to vary, with optimism and life satisfaction generally similar (except in relation to parental education). Whites reported the greatest optimism and satisfaction, whereas Blacks reported the greatest positive affect. The highest optimism and satisfaction levels were evident among college-educated participants, but positive affect was not associated with educational attainment. Consistent with other studies, optimism was also greatest among participants with at least one college-educated parent [52,53], although this association was of a smaller magnitude than the other statistically significant findings. In contrast, life satisfaction and positive affect were not linearly associated with parental education.

We also found that optimism and life satisfaction were patterned by occupational class such that individuals with professional or managerial jobs were the most optimistic and satisfied. Occupational prestige also had a clear gradient with optimism and satisfaction whereby more prestige correlated with more optimism and satisfaction. This was not the case with positive affect, which conflicts with previous findings (but this could be due to differences in methodology including single sex samples and ecologic momentary assessment methods) [3]. Finally, consistent with past work [54,55], optimism and satisfaction each demonstrated a positive, linear association with income. Although we did not find a statistically significant association between positive affect and income, previous studies report small associations and suggest income’s relation with affect is smaller and more unstable than with satisfaction [56].

When examining intergenerational social mobility, the most optimistic and satisfied individuals were those with high levels of education across generations (i.e., parent and participant were both college-educated). These findings are consistent with those from Heinonen and colleagues [11] who studied Finnish youth into adulthood, although they also found that the downwardly mobile were more optimistic than the upwardly mobile and the persistently low. In our sample of mostly middle-aged U.S. adults, those who were upwardly mobile were more optimistic and satisfied than the downwardly mobile. We also found that individuals with persistently high levels of education across generations did not differ in positive affect from those with persistently low levels of education, the upwardly mobile, or the downwardly mobile. To our knowledge, only one other study has examined links between satisfaction and social mobility [57], and no studies have examined positive affect.

Dispositional optimism, life satisfaction, and positive affect are sometimes used as interchangeable markers in studies examining psychological factors in relation to physical health. Our findings suggest that such practices should be avoided, particularly if studies are investigating a possible role for psychological assets in explaining social disparities in physical health. These measures are not proxies for one another as they have different associations with SES. Optimism was consistently related with structural factors, which is in line with the reserve capacity framework. Namely, higher levels of dispositional optimism were evident among more advantaged members of society: white, college-educated individuals with higher occupational positions, prestige, and income. Satisfaction had a similar pattern, with the exception of parental education. Compared with optimism and satisfaction, positive affect seemed to have unique associations with status [56]. This could be because positive affect taps the emotional aspect of well-being whereas optimism and satisfaction are cognitively-oriented. Alternatively, perhaps because affect has evolved to signal whether a particular stimulus may be beneficial or harmful, the utility of positive affect may cut across different levels of SES. Moreover, optimism and satisfaction may be patterned similarly because they reflect more enduring characteristics compared with positive affect, which may be subject to transient influences. Or, optimism’s (and to a lesser extent satisfaction’s) emphasis on fulfilling meaningful goals versus positive affect’s emphasis on enjoying pleasure may be more strongly tied to social structural status and capacity to attain goals. Having the educational, financial, and other resources associated with higher status may further enable greater striving and more hopefulness about the future. Although these speculations extrapolate beyond the current data, they could be investigated in future research designed to examine the association between psychological assets and structural factors implicated in opportunities (or the lack thereof) across the life course.

The correlational design of the current study prohibits causal conclusions and the size of the reported associations could be considered small according to conventional guidelines. However, reported associations are comparable to other findings [58] and even very small effects can have a large impact at the population level [59], especially if effects stemming from SES and psychological assets accumulate across the life course [60]. This study captures structural factors at only a single point in time, so it is unclear whether a recent change in status (e.g., changing from employed to unemployed) is associated with changes in psychological assets. In addition, educated individuals were more likely to participate in MIDUS II [34], so reported associations may be conservative due to selective attrition. Although study participants ranged in age from 30–85 years, most were middle aged. And, despite supplementing the sample with Blacks, the current sample was primarily White and numbers were not large enough to investigate other racial or ethnic groups separately. Generalization to more diverse samples may not be warranted, although evidence suggests that optimism and other psychological assets are universal phenomena [61,62]. In addition, findings related to intergenerational social mobility were based only on education, not other status indicators such as employment status, occupational class, or income [63,64]. Moreover, educational attainment in earlier generations may not correspond exactly to education attainment in subsequent generations (e.g., a high school diploma for somebody in the early 20^th^ century may be equivalent to a college degree for somebody in the mid to late 20^th^ century).

Despite these limitations, this is one of the first studies to broadly examine dispositional optimism in relation to one’s socioeconomic position among mostly middle-aged U.S. men and women. Findings indicate that optimism is contoured by social structural factors related to opportunity (i.e., race/ethnicity, education, occupational class and prestige, income, and social mobility), perhaps because such factors provide the context in which optimistic tendencies are shaped, developed, or used. Although associations between structural factors and optimism at first appear modest, they may have meaningful implications at the population level or as effects accumulate across the lifespan. Indeed, the 4-point spread in optimism scores evident for the highest versus lowest levels of participant education has been shown to translate into a 16% reduced risk of myocardial infarction and a 30% reduced risk of heart disease-related mortality [9]. Thus, even apparently small differences in optimism can have critical health implications.

We also compared the patterning of optimism with two other widely assessed psychological assets, which has not been done previously. Although dispositional optimism is not routinely assessed in surveillance studies, findings reported here suggest that it may reveal unique insights about effects of social structure on health (among other pathways), and should be considered for inclusion in future assessments. Moreover, given links between optimism and improved mental and physical health [6,65], assessment of optimism may provide additional insight into social disparities in health or other factors that shape capacity for positive adaptation in the context of adversity [66]. Because optimism and satisfaction appear to be related to social structural influences and health, they may be useful targets for both policy-oriented as well as individual-level intervention strategies for improving population health. Furthermore, increasing educational opportunities and otherwise reducing social disparities may not only improve physical health, but may also foster greater psychological assets.
